# Repeated, Selection-Driven Genome Reduction of Accessory Genes in Experimental Populations

**DOI:** 10.1371/journal.pgen.1002651

**Published:** 2012-05-10

**Authors:** Ming-Chun Lee, Christopher J. Marx

**Affiliations:** 1 Department of Organismic and Evolutionary Biology, Harvard University, Cambridge, Massachusetts, United States of America; 2 Faculty of Arts and Sciences Center for Systems Biology, Harvard University, Cambridge, Massachusetts, United States of America; Yale University, United States of America

## Abstract

Genome reduction has been observed in many bacterial lineages that have adapted to specialized environments. The extreme genome degradation seen for obligate pathogens and symbionts appears to be dominated by genetic drift. In contrast, for free-living organisms with reduced genomes, the dominant force is proposed to be direct selection for smaller, streamlined genomes. Most variation in gene content for these free-living species is of “accessory” genes, which are commonly gained as large chromosomal islands that are adaptive for specialized traits such as pathogenicity. It is generally unclear, however, whether the process of accessory gene loss is largely driven by drift or selection. Here we demonstrate that selection for gene loss, and not a shortened genome, per se, drove massive, rapid reduction of accessory genes. In just 1,500 generations of experimental evolution, 80% of populations of *Methylobacterium extorquens* AM1 experienced nearly parallel deletions removing up to 10% of the genome from a megaplasmid present in this strain. The absence of these deletion events in a mutation accumulation experiment suggested that selection, rather than drift, has dominated the process. Reconstructing these deletions confirmed that they were beneficial in their selective regimes, but led to decreased performance in alternative environments. These results indicate that selection can be crucial in eliminating unnecessary genes during the early stages of adaptation to a specialized environment.

## Author Summary

Many free-living bacteria are known to commonly lose large portions of their genomes, but it is unclear what evolutionary forces drive these changes. Is this due to random loss in small populations, as is thought to be the case for the extreme genome degradation of intracellular symbionts, or due to selection? And if it is beneficial, is it directly caused by replicating a shorter genome or advantageous loss of the genes themselves? We uncovered that most replicate populations of *Methylobacterium extorquens* AM1 evolved in the laboratory for 1,500 generations lost nearly 10% of their genome. Through reconstructing these deletions, we demonstrated that these losses were indeed beneficial, but the advantage did not scale with length of genome lost, and were even deleterious in alternative environments. These findings suggest that the initial stages of genome shrinkage may be driven by selection, ultimately leading to a more streamlined, specialized organism.

## Introduction

Bacterial genomes have the potential to rapidly change their size and content as a result of various mechanisms such as deletion, duplication and horizontal gene transfer. The net expansion or contraction at the genome scale is thus a function of both the rate at which these events occur and the subsequent filters imposed by natural selection and/or genetic drift [1], [2]. Although most bacterial genomes have remained relatively constant in size due to an apparent overall balance of these forces [3], distinct strains within a species can differ remarkably in gene content [4]. This finding has led to categorizing the genome into the core and accessory (or auxiliary) components, the former being present in nearly all members, and the latter being present in only a subset of strains [5].

The population biology and selective environment of microbes each contribute to the tempo and mode of genomic change. Of primary importance is the effective population size (*N_e_*) of a species, as this influences the efficacy of selection versus drift. Repeated bottlenecks, such as those experienced by intracellular endosymbionts (which also participate in little, if any horizontal gene transfer), result in tremendous rates of sequence change and ineffective selection to maintain functions required for host-independent lifestyle. This often leads to loss of many genes that are essential for the free-living microbes and massive genome shrinkage (ex: 77% in the intracellular symbiont of aphids, *Buchnera aphidicola* and genomes as small as *Hodgkinia cicadicola* (144 kb)) [2], [6]–[9]. On the other hand, simply living on a restricted set of resources in a relatively constant environment can also result in reduced genomes despite very large *N_e_*, such as observed for the plankton *Prochlorococcus* and *Pelagibacter* [10], [11]. For these it has been suggested that the major force driving genome reduction is streamlining, defined as when “selection acts to reduce genome size because of the metabolic burden of replicating DNA with no adaptive value” [11]. In addition to DNA synthesis, deletions also eliminate producing the RNA molecules and proteins encoded by that region.

Beyond external factors, the genomic structure of microbes and mechanisms of gene gain and loss make it possible for large regions to come and go in single events. Accessory genes are disproportionately found on extrachromosomal replicons that are subject to potential loss. Alternatively, even when present on the main chromosome accessory genes are often found as discrete genomic islands disrupting an otherwise syntenic chromosome between strains in a species. This can result in gains or losses via various mechanisms such as homologous or site-specific recombination and phage integration/excision [12], resulting in punctuated large-scale gene content changes. Large-scale reductions of accessory genomes via these events may be a critical mechanism in early stages of genome shrinkage.

Although either drift or selection could contribute to genome reduction observed in nature, we lack direct evidence to distinguish between the lack of purifying selection to maintain the genes lost versus positive selection for their loss. Genomic analyses of chronic infections, such as *Pseudomonas aeruginosa* in cystic fibrosis patients, have repeatedly observed large deletions [13]–[15]. This rapid loss of genomic islands could simply be due to high rates of recombination and drift (or hitchhiking). Alternatively, the instability of the accessory genome could be due to selection, either for reduced genome length (*i.e.*, streamlining) or beneficial gene loss, such as has been shown for *Shigella flexneri*, a facultative intracellular pathogen of primates [16].

Laboratory-evolved populations of bacteria present the unique opportunity to address the forces involved in genome reduction under selective regimes that tilt the relative efficacy of selection versus drift. ‘Mutation accumulation’ experiments purposefully use single-colony bottlenecks at each transfer to maximize drift [17]. In contrast, the more typical experimental evolution regimes maintain an *N_e_* often in the millions, allowing selection to dominate [18], [19]. To date, genome reductions found in the above experiments have tended to be modest (up to 4% of the genome in mutation accumulation experiments, and 1% in larger populations). The DNA loss rates observed have been low (∼2 bp per generation), and the regions lost have largely been inconsistent across lineages. With the exception of small (1.6–7 kb) deletions in the ribose gene cluster of *Escherichia coli* [20], none of these genome reductions have been tested for their fitness effects. As such, it remains unclear whether these observed genome reductions imparted an advantage in the selective environment, whether fitness effects scale with the length of DNA removed, and/or whether such events generate tradeoffs across other environments.

Here, we used experimental evolution to investigate the role of large-scale deletions in adaptation and specialization. We evolved populations of the α-proteobacterium *Methylobacterium extorquens* AM1, a member of the dominant genera found on leaf surfaces [21], [22]. Like other bacteria that utilize single-carbon (C_1_) compounds (*e.g.*, methanol) as growth substrates, *M. extorquens* AM1 has also specialized to grow on a very limited array of multi-C compounds (*e.g.*, succinate), and has been a model for exploring rapid metabolic specialization during adaptation [22]. Across 32 populations evolved for 1500 generations in one of four different nutrient regimes we found 80% of these deleted the same genomic region that encompasses up to 10% of the genome. By reconstructing these deletions under the ancestral genetic background we have demonstrated that they rose in frequency due to selection; however, the advantage gained was not a generic effect of shortening genome length, but was specific to the region lost and imparted an advantage (or disadvantage) that depended upon the environment.

## Results/Discussion

### Adaptation of populations and identification of massive, parallel deletions

In order to examine the potential role of large-scale deletions in adaptation of *M. extorquens* AM1 we analyzed genome content from replicate evolved populations. Eight parallel populations were grown at a large *N_e_* (∼2.5×10^8^) in each of four different nutrient regimes (32 populations in total): methanol, succinate, mixture of methanol and succinate, or alternating between methanol and succinate. After 1500 generations, the evolved populations increased fitness in their selective environments by 15 to 37% compared to their wild-type ancestor (Figure S1). As reported previously [22], a couple of these strains were actually less fit than the ancestor, which likely represent genotypes that exist due to frequency-dependent interactions such as cross-feeding. For comparison, we also maintained 10 lineages on solid medium for 1500 generations that we transferred through single-cell bottlenecks to maximize the strength of drift.

**Figure 1:**
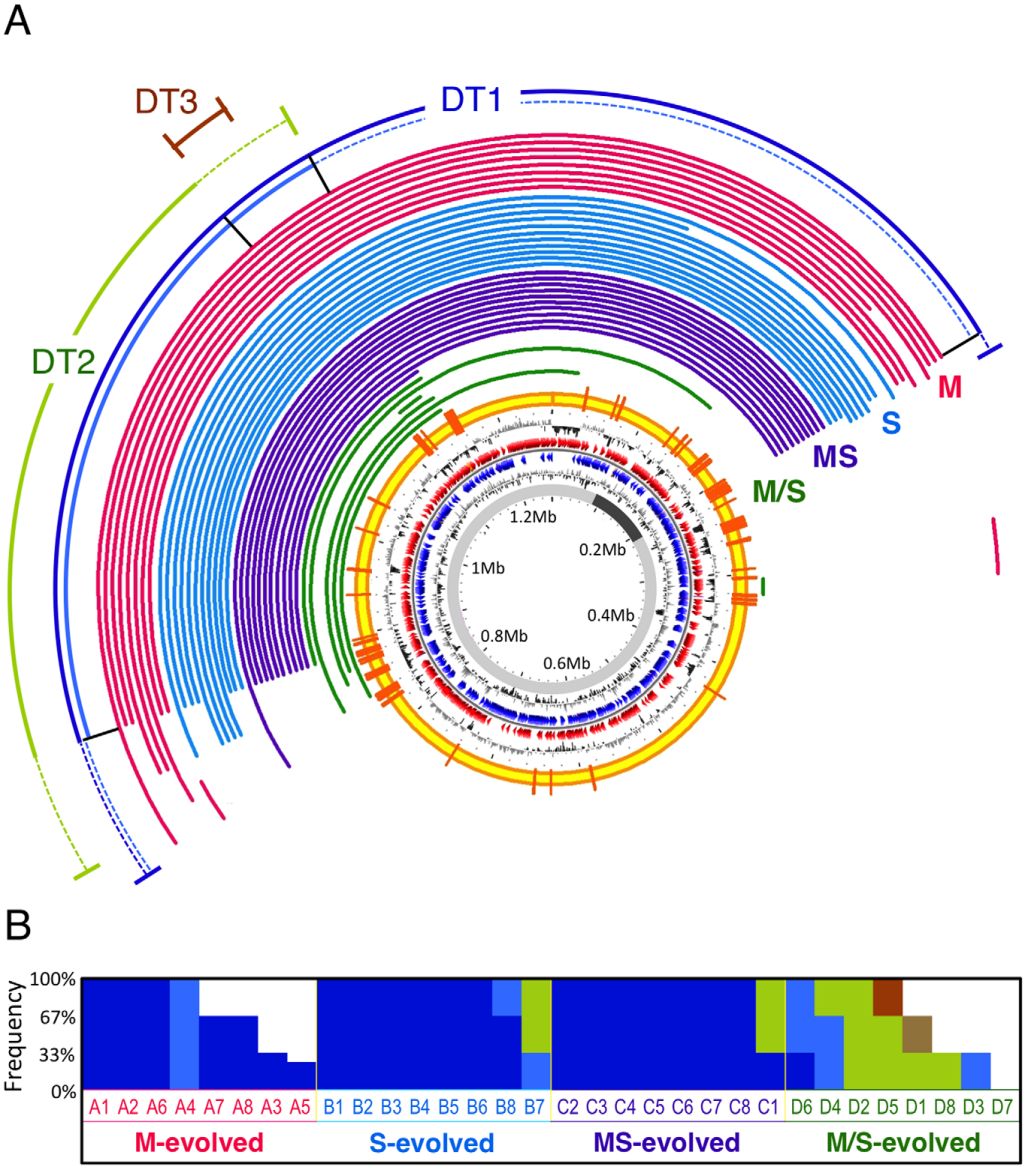
Parallel deletions on megaplasmid found in evolved isolates. (A) Deletions detected by CGH arrays. Each arc represents the deleted region in an isolate with the color indicating the selective environment: pink, M (methanol); blue, S (succinate); purple, MS (methanol+succinate); green, M/S (alternating methanol/succinate). Observed deletion types (DT1, 2, and 3) were classified by PCR detection using 4 pairs of primers in region R1, R2, R3 or R4 (black bars from left to right) and are shown as the outside arcs with dashed lines indicating the ranges of edges for subtypes. DT1a: all 4 pairs gave negative results (dark blue). DT1b: R1, R2, and R3 negative R4 positive (light blue). DT2: R1 and R2 came back negative but R3 and R4 were positive (light green). DT3a: only one isolate had this deletion, which was detected by the arrays (brown). DT3b: R1, R2 and R4 came back positive but R3 was negative (not shown in Figure 1A). (Methods, Tables S1 and S3). Successive circles from inside to outside: conserved region in *M. extorquens* DM4 and CM4 (dark grey), GC skew, predicted CDSs transcribed in the counterclockwise direction (blue), predicted CDSs in clockwise direction (red), GC% deviation, IS position (orange bar). (B) Frequency of deletion types in isolates from populations. Three or four isolates were obtained in each population (labeled columns sorted by number and type of deletions for clarity) and their deletion type is indicated by color: DT1a, dark blue; DT1b, light blue; DT2, light green; DT3a, brown; DT3b, light brown.

To determine the extent to which large-scale deletions contributed to adaptation, we used comparative genomic hybridization (CGH) to uncover chromosomal changes in 44 isolates from the 32 evolved populations (Table S1). Like many bacteria, the 6.9 Mb genome of *M. extorquens* AM1 has multiple replicons of varying sizes (5.5 Mb chromosome, 1.3 Mb megaplasmid present at one copy per chromosome, and 3 plasmids between 25–44 kb present between 1–3 copies per chromosome) [23], a total of 23 distinct deletions were identified, some of which in more than one lineage (Table S2). Over 91% of the deletion events were due to homologous recombination between matching sequence regions, and of these, 86% were between co-directional pairs of one of the 142 insertion sequences (ISs) present in the genome of *M. extorquens* AM1 [23]. Most notable were the extensive, repeated changes to the megaplasmid: 36 of the 44 isolates screened by CGH contained deletions spanning a single region that ranged from 23 kb to 641 kb (Figure 1A). The largest of these deletions removed 24.7% of the accessory genes (unique to *M. extorquens* AM1 versus strain DM4) [23] and 2.7% of shared, core genes. This represents the largest parallel losses observed during laboratory adaptation thus far. Previous experiments either observed an occasional large deletion (200 kb) [17] or repeated loss of small regions (<7 kb) [20]. Applying a PCR-based screen to 56 additional isolates revealed 51 more with deletions in this region (Table S1). Despite this overall parallelism, the precise borders of these deletions were somewhat different. We broadly classified these into three classes of deletion types (DT1, 2, and 3; Figure 1A). A DT1 event with borders precisely at a co-directional pair of ISs had been independently identified by genome re-sequencing of a methanol-evolved isolate from a population initiated with a different starting genotype [24]. These deletion types were present at significantly different proportions across the four nutrient regimes (Figure 1B). Moreover, since distinct subtypes of deletions coexisted in some populations at changing frequencies (Figure 1B and Text S1), the larger deletions may have occurred stepwise, as proposed for similar events in the genomes of *B. aphidicola* strains [25].

### Large-scale deletions were beneficial in the selective environments they arose

The observed parallelism across replicates could be due to either an unusually high rate of occurrence and/or a selective advantage conferred by the events themselves. For example, the parallel deletions of regions of the ribose operon of glucose-evolved *E. coli* were shown to partly depend upon a high rate of transposition and subsequent recombination [20]. To address this possibility, we examined the 10 populations transferred through single-colony bottlenecks for 1500 generations. None of the defined deletion types were detected by PCR, which is significantly unlikely to be observed given the rate they appeared in the large *N_e_* populations (P<0.0001) (Text S1).

**Figure 2:**
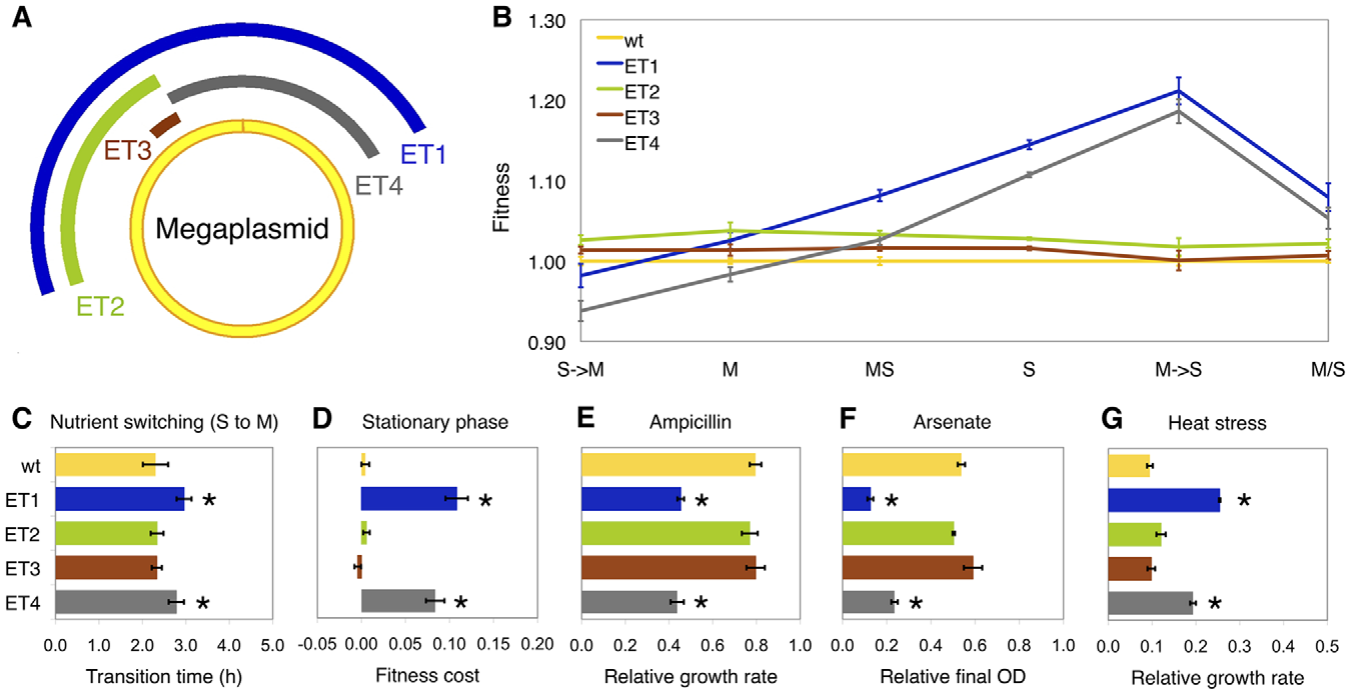
Phenotypes of deletion mutants. (A) Schematic view of engineered deletion mutants. Each arc represents the deleted region in the mutant ET1 (dark blue), ET2 (green), ET3 (brown) and ET4 (grey). (B) Reaction norms of fitness for deletion mutants and wild type in 4 selective environments: M, S, MS, M/S, and each half-environment of M/S (M→S and S→M). (C) Transition time from S to M. (D) Fitness cost at stationary phase estimated as the fitness drop from hour 28 to hour 48. (E–G) Succinate-grown cultures with the following treatments: E, ampicillin (12.5 µg/mL); F, arsenate (30 mM); G, 36°C. Relative growth rate or final OD_600_ (optical density) was calculated as the ratio of with and without treatment. Error bars represent 95% confidence intervals and significant differences from wild-type are indicated by *(P<0.05).

In order to directly test for a possible selective advantage of these deletions, we reconstructed deletions in the wild-type ancestor and tested whether these were individually beneficial in their selective environments. We created four deletions that represent the largest class found (engineered type 1, ET1), the half of ET1 that was commonly lost (ET2), a small region, itself only observed once, at the intersection of all identified deletions (ET3), and a fourth region (ET4) that, although never observed to be lost in the evolved populations, removed the alternative half of DT1 and was equivalent in length to ET2 (both ∼300 kb) (Figure 2A). With the exception of ET3 in the methanol/succinate switching environment, all deletion types were individually beneficial in the selective environments that they were observed in, with up to a 15% selective advantage for ET1 in succinate medium (Figure 2B). The nearly neutral fitness effect of ET3 indicated that the beneficial effect was not due to removing this shared region. Interestingly, the fitness effect of ET1 was approximately the same as expected from the two half deletions (ET2 and ET4), suggesting that there is little epistasis between these two regions (Figure S2 and Text S1).

### The selective advantage of gene loss is not due to a shorter genome and leads to tradeoffs in alternative environments

Two lines of evidence refuted the hypothesis that the physiological basis of the fitness advantage of the large-scale deletions was simply due to a shorter genome, and rather suggested that loss of specific gene(s) was the primary benefit. The streamlining hypothesis that genome reduction is driven by metabolic efficiency of a shorter genome would predict that: 1) the magnitude of benefit would scale with size of the deletion and 2) the benefit would be reasonably similar across multiple environments. First, we found that selective advantage did not correlate with deletion size. This is most clearly demonstrated by comparing ET2 and ET4, which have equivalent lengths. These two ∼300 kb deletions exert quite different effects, whereas ET1 (which is twice as large) and ET4 behaved quite similarly. Second, we found that, although the marginal benefits of ET2 and ET3 were relatively constant across different growth substrates, but the phenotype of ET1 and ET4 varied markedly. This included being a disadvantage during growth on methanol when transferred from succinate, which appears to be due to a longer transition time between nutrients and decreased fitness during stationary phase on succinate (Figure 2C, 2D and Figure S3). This result is in consistent with a recent report where no correlation between genome size and selection intensity was found across a variety of natural isolated bacteria [26]. The high prevalence of observing DT1 in populations evolved in succinate and the methanol-succinate mixture is in accord with the above phenotypes, but other factors such as epistatic interactions with previous mutations may account for the surprisingly high frequencies of DT1 in methanol and low frequency in methanol/succinate switching environments. Given that the various deletions appeared (above the limit of detection) in the second half of this 1500 generation experiment, other mutations would have already been present that may alter the selective effect of these losses (Text S1).

Although the large-scale deletions from the megaplasmid of *M. extorquens* AM1 were beneficial in the laboratory environment, further tradeoffs suggest that loss of this region would have consequences in natural environments. Unlike regular plasmids, megaplasmids and minichromosomes (or ‘chromids’) are long-term replicons residing in more than 10% of bacterial genomes across markedly different life styles [27]. The GC%, coding density and the percentage of repeat region of the megaplasmid in AM1 are compatible with the main chromosome but very different from the other 3 small plasmids present in this genome, indicating its long-term existence in this strain. We first examined the potential functions of the megaplasmid by COG analysis, which showed an overrepresentation of genes related to metabolic functions in deleted regions (*X* ^2^ test, *P*<0.0001, Figure S4). This is consistent with the observation of other reduced genomes [7]. Furthermore, the predicted functions of the genes encoded within the deleted regions were consistent with a lack of essentiality due to duplicate copies of potentially essential genes on the main chromosome. Indeed, with a fairly liberal definition of homology (minLrap≥0.8; maxLrap≥0; identity≥60%) there are 159 genes on the megaplasmid that have the homologs on the main chromosome. As 113 of these are found in DT1, a significant overrepresentation relative to the rest of the megaplasmid (*X* ^2^ test, *P*<0.0001), and all regions with synteny with the chromosome for ten or more genes are located in the deleted region, some of the benefit may have come from removing these possible redundancies. On the other hand, many genes putatively involved in stress responses would have been lost (Table S5). We therefore tested the deletion types across a panel of stresses, revealing that ET1 and ET4 had decreased resistance to ampicillin and arsenate (Figure 2E and 2F), and increased growth at the upper end of the temperature range of *M. extorquens* AM1 (Figure 2G). The loss of two sigma factors (Figure S5) and genes shown to be involved in leaf surface colonization [28] by these deletions lends further support that some of these genes contributed to the ecology of this strain.

### Conclusions

These data have provided a rare opportunity to demonstrate that selection for gene loss contributed to the repeated, large-scale removal of accessory functions from adapting genomes. The selection regime we applied was a seasonal environment of growth and starvation, but since only one or two supplied resources and all other environmental factors were held constant, this rendered many functions unnecessary. It is quite common for plasmids bearing antibiotic resistance genes or toxins to be lost when these functions go unrewarded; however, this process differs substantially what is described here in terms of the scale of genome change, the presence of genes that would be essential if it were not for a duplicate copy, as well as the mechanism of loss (unfaithful segregation vs. homologous recombination).

Given that so many genes were lost in these deletion events, future work will be required to pin down whether few or many genes contribute to the observed phenotypes and by how much. Numerous stress response genes were lost in these events, and it has been in multiple cases that there can be tradeoffs between growth capacity and stress response in environments ranging from chemostats [29] to long-term stationary phase [30]. Similarly, the deletions that sped growth on most substrates led to an impaired capacity to deal with nutrient switches, starvation, and the toxic effects of an antibiotic and a toxic metal. Although these large-scale losses were successful due to the benefit they conferred in the flask, it is quite likely that they would impart tradeoffs in components of the natural environment inhabited by *Methylobacterium*.

Selection-driven loss of accessory genes can rapidly limit the niche of a given lineage, resulting in restricted lifestyle and lowering both *N_e_* and access to horizontal gene transfer with other members of the species. Indeed, aspects of our laboratory conditions and starting strain - a sudden restriction in niche breadth and now unnecessary accessory functions present in contiguous islands - commonly occurs in natural environments, such as the establishment of chronic infections by opportunistic pathogens where analogous deletion events have been identified [13]–[15]. Smaller, more isolated populations in which purifying selection for previously useful functions is absent can lead to further genome reductions as drift becomes increasingly relevant. Thus, although in other scenarios bottlenecks leading to loss of functions via drift could initiate specialization, our results emphasize the potential for selection-driven, large-scale deletions of unnecessary genes as a route towards a limited niche and the beginning of a path leading to further genome changes.

## Materials and Methods

### Experimental evolution populations

This paper examines isolates from 32 populations that were founded from two nearly isogenic strains of wild-type *Methylobacterium extorquens* AM1, CM501 and CM502, which have pink and white colony color, respectively [31]. These populations evolved in four different environments each with 8 replicates (odd numbers founded by CM501; even by CM502): methanol (M, 15 mM, ‘A’ populations), succinate (S, 3.5 mM, ‘B’ populations), a mixture (MS) of methanol (7.5 mM) and succinate (1.75 mM) (‘C’ populations), and alternating (M/S) between methanol (15 mM) and succinate (3.5 mM) (‘D’ populations). The general selective regime, minimal medium and culturing conditions utilized were described previously along with the initial examination of the dynamics of adaptation and specialization of the A and B populations [22]. The C and D populations were evolved in the same conditions except for the mixed or alternating substrate conditions. Briefly, populations were grown in 9.6 mL of medium and cultured at 30°C in 50 mL flasks with 225 rpm shaking. Serial transfers were performed every 48 hours using 1/64 dilutions (*i.e.*, 6 generations) with a population size at the end of each cycle of ∼2×10^9^. Three or four evolved isolates were obtained from generation 1500 of each population with preference for different colony morphologies, where apparent. From each population, one or two isolate(s) were chosen to test in genomic microarray analysis, and the remaining colonies were screened for deletions via PCR (Table S1).

### Mutation accumulation system

Ten mutation accumulation lines were founded by CM501 and prorogated at 30°C on solid media comprised of half nutrient agar and half ‘hypho’ agar containing succinate (7.5 mM final concentration) [31] to allow rapid colony formation. For each lineage, every 3.5 days the last colony on the streak line was picked as a random sample and streaked on a new plate. The population expanded from one cell to approximately 10^6^ cells in a colony each passage, representing ∼20 generations, and was repeated 75 times (∼1,500 generations).

### Deletion detection via comparative genomic hybridization (CGH)

DNA isolation was performed using the Wizard Genomic DNA Purification Kit (Promega, Madison, WI) following the manufacturer's protocol. Deletions in evolved strains were identified using comparative genomic hybridization arrays performed by MOgene Inc. (St. Louis, MO), a certified Agilent service provider. The custom arrays spotted with the ancestor genome were designed, printed, and probed as described [32]. Without the necessity to detect quantitative signals, each sample was labeled with either Cy3 or Cy5 and hybridized once with a sample labeled with the other dye. In total, 25 hybridizations were done for 45 samples (44 evolved strains and the ancestor), including three control experiments (Table S2).

### Deletion confirmation by PCR and sequencing

To confirm each deletion, one primer outside (p1 & p4) and inside (p2 & p3) that region was designed for each side. The deletion was confirmed if fragment was amplified by p1 & p4 but no product was amplified by p1 & p2 or p3 & p4. For fragments shorter than 1 kb, exact junctions were verified via sequencing. Products longer than 1 kb were analyzed via restriction digests to compare with the predicted patterns from the genome sequence. All confirmed deletions were consistent with array results. The precise junctions of all deletions on the main chromosome were identified except two of the deletions in CM1055 and CM1820 due to the presence of multiple repeat elements around their flanking regions. For the deletions on the megaplasmid, we only focused on the parallel pattern of the deletions and did not confirm each various subtype with their slightly different endpoints.

For detecting deletions in isolates not screened via CGH, we designed 4 pairs of primers to amplify regions across DT1, each with upstream and downstream pairs (Table S3). We classified isolates into 4 major types based on the PCR results (DT1 with 2 subtypes): DT1a (negative results from all), DT1b (negative result from R1, R2, and R3 and positive result from R4), DT2 (negative result from R1&R2 and positive result from R3&R4), DT4 (positive result from R1&R2 and negative result from R3&R4; not found in any population), and no deletion (positive results from all). The deletion in CM1194 was categorized as DT3a based on the array data; the deletion in CM1182 was categorized as DT3b based on the negative result from R3 but positive results from the other 3.

### Construction of deletion mutants

Allelic exchange plasmids for generating deletion mutants were constructed based on pCM433, a *sacB*-based suicide vector [31]. PCR products of regions upstream and downstream of each deletion were amplified and consecutively cloned into pCM433 to generate pML4, pML5, pML7 and pML9 (Table S4). In order to reduce false-positives, a second selection marker, *kan*, with *loxP* excision sites amplified from pCM184 [33] was introduced into each of the plasmids between the upstream and downstream regions to generate pML10, pML11, pML12 and pML13, respectively (Table S4). Each of these donor plasmids was then introduced into the wide type strain (CM501) via triparental conjugations as previously described [31]. Single-crossover recombinants were selected with tetracycline (Tet, 10 µg mL^−1^) and then double-crossover recombinants were selected with kanamycin (Kan, 50 µg mL^−1^) and sucrose (5% wt/vol.). For each deletion type, we saved three independent clones through the cloning steps. These were each confirmed by PCR to contain the correct deletion and all three were tested for a consistent phenotype. The *kan* marker was then excised by *cre* recombinase as before [33] to generate the desired unmarked deletion mutants (Table S1).

### Growth and fitness assays

We performed the growth and fitness assays following a previously described procedure [22] with a few modifications. Briefly, three replicate cultures of each strain were inoculated and acclimated in minimal medium supplemented with carbon sources in 48-well plates (Corning, Lowell, MA) at 30°C, 650 rpm, 1 mm orbit and a total volume of 640 µL in each well. Growth curves were then obtained by following the change in OD_600_ (Victor^2^ plate reader, Perkin Elmer, Waltham, MA). The transition time between growth phases observed during growth on MS (M∶S = 7∶1) was estimated as before [34]. Growth rates and regression lines for each phase were calculated (Phase I: y = a_1_+b_1_x; Phase II: y = a_2_+b_2_x), the OD_600_ at the time of transition (OD_t_) was determined as the average of two OD_600_ values with the minimum change during the transition phase, and the effective transition time was obtained as the difference between the two time values (x_1_, x_2_) where the estimated regression lines were equal to OD_t_.

Fitness of each strain was measured as before [22] by competing each evolved or constructed strain against a fluorescently labeled ancestor (CM1179) strain in 48-well plates with initial volumetric ratio of 1∶1. Due to the small fitness changes for certain strains, competition assays were run for 4 cycles of growth (*i.e.*, 8 days). The ratio of non-fluorescent cells in mixed populations was measured by passing population samples before (R_0_) and after 4 cycles of competition growth (R_4_) through a BD LSR II flow cytometer (BD Biosciences, San Jose, CA). Fitness values (W) were calculated by following equation:
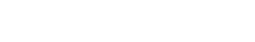
To estimate the fitness cost on succinate during stationary phase, cultures were also sampled at hour 28 (early stationary phase), such that the fitness cost was estimated as the difference in fitness values calculated between hours 0 to 28 vs. 0 to 48 (using 9 replicates per strain).

### General stress response assays

Disc diffusion assays were done to test for sensitivity on formaldehyde, SDS, peroxide, a trace metal mix, salt, arsenate and amplicillin. Bacteria were grown to stationary phase (OD_600_∼1.5) in regular hypho medium supplied with 3× succinate (10.5 mM) . Five mL of this culture was mixed with 60 mL of 42°C pre-warmed soft agar (0.75%, with 15 mM succinate), and 5 mL of this mixture was poured onto hypho agar plates with 15 mM succinate. Disks were placed at the center of the plates and aliquots (5 µL) of formaldehyde (37%), SDS (10%), peroxide (30%), a trace metal mix (1000×) (Delaney et al. unpublished), NaCl (1 M), sodium arsenate (10% w/v) or amplicillin (100 mg/mL) were added on the filter discs. Diameters of growth inhibition were measured after 4 days.

Exponential-phase cells growing on succinate (OD_600_∼0.5) were used in heat shock and UV resistance assays. Cells were transferred to 55°C for 15 min for heat shock or exposed to 312 nm UV light for 15 min for UV resistance assays. Suspensions were then diluted and plated onto hypho agar containing 15 mM succinate, and colonies were counted after 4 days of 30°C incubation.

Additionally, succinate-grown cultures were tested in liquid medium with the following treatments: formaldehyde (1–5 mM), SDS (10^−1^–10^−5^‰), peroxide (10^−1^–10^−5^‰), trace metal mix (2–20×), salt (5–500 mM), ampicillin (12.5–50 µg/mL), sodium arsenate (20–50 mM), UV exposure prior to growth (1–20 min), or heat stress during growth (32–36°C). Final OD_600_ or relative growth rates were calculated as the ratio of treatment to control.

## Supporting Information

Figure S1 Average fitness increase of evolved populations in their selective environments. The boxplot shows the mean and variation of 25 isolates in each environment. Average fitness increases are 14.5%, 26.0%, 20.7%, 24.8% for A (M), B (S), C (MS) and D (M/S) populations, respectively. As reported previously [22], a couple of these strains were actually less fit than the ancestor, which likely represent genotypes that exist due to frequency-dependent interactions such as cross-feeding.
(TIF)

Figure S2 Relative growth rates and additive effects in fitness values of ET2 and ET4 in various environments. Dots represent the relative growth rates of mutants in all 5 environments, calculated as the ratio to the wild-type. Error bars represent 95% confidence intervals. Similar patterns were found as in fitness values except in S→M and M→S where the fitness of ET1 and ET4 change significantly but the growth rates remain the same, when compared to the corresponding environment (M and S, respectively). The light blue dash line represents the product of fitness values for ET2 and ET4, which is not significantly different from the fitness values of ET1 in all environments. The result indicates ‘non-epistatic’ interaction between ET2 and ET4; the proportional effect of ET2 and ET4 is unchanged when present together in ET1.
(TIF)

Figure S3 Viable counts of selected deletion mutants in 4 environments (M, S, M→S, S→M). All mutants and wild type have similar viable counts even after 96 hours. Less than 15% drop of viable counts was observed for all mutants.
(TIF)

Figure S4 Functional analysis of DT1 versus undeleted region on the megaplsmid. The number of ORFs for each major functional category was calculated based on COG classification. There are substantially more metabolic related genes in DT1 region than the undeleted region.
(TIF)

Figure S5 Phylogeny of sigma factors in *M. extorquens* AM1 (META1, pink text; META2, orange text) and closely related strains: *M. extorquens* DM4 (METDI), CM4 (Mchl), PA1 (Mext), *M. populi* BJ001 (Mpop). Five groups of sigma factors are colored based on the annotation. Arrows indicate the sigma factors which locate in the region of ET2 (green) and ET4 (grey). META2_0154 (ECF type) and META2_0121 (sigma 24) are conserved in all *M. extorquens* strains, suggesting a potential function of those two sigma factors.
(TIF)

Table S1 *Methylobacterium* strains used in the study.
(DOCX)

Table S2 Deletions detected by CGH arrays.
(DOCX)

Table S3 Primer list.
(DOCX)

Table S4 Plasmid list.
(DOCX)

Table S5 Functional gene list within deletion region.
(DOCX)

Text S1 (DOCX)
