# Supplementary figures and images for: Repeated, Selection-Driven Genome Reduction of Accessory Genes in Experimental Populations
Source: PLoS Genet. 2012 May 10;8(5):e1002651. doi: 10.1371/journal.pgen.1002651 (PMC3349727; doi:10.1371/journal.pgen.1002651)

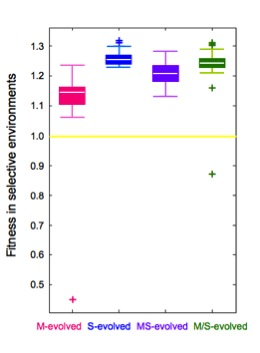

Supplement: Figure S1 — Average fitness increase of evolved populations in their selective environments. The boxplot shows the mean and variation of 25 isolates in each environment. Average fitness increases are 14.5%, 26.0%, 20.7%, 24.8% for A (M), B (S), C (MS) and D (M/S) populations, respectively. As reported previously [22], a couple of these strains were actually less fit than the ancestor, which likely represent genotypes that exist due to frequency-dependent interactions such as cross-feeding. (TIF) [file pgen.1002651.s001.tif]

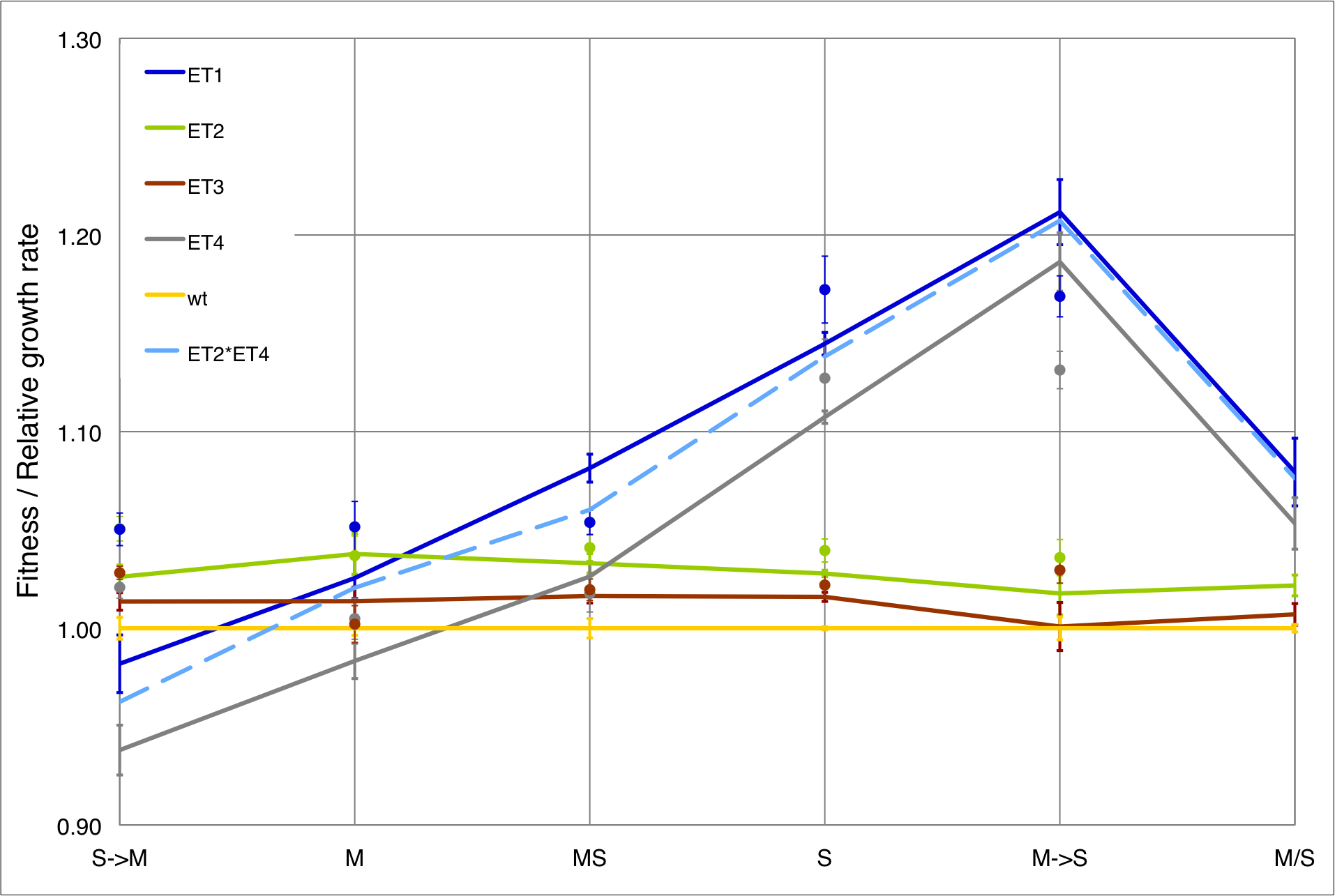

Supplement: Figure S2 — Relative growth rates and additive effects in fitness values of ET2 and ET4 in various environments. Dots represent the relative growth rates of mutants in all 5 environments, calculated as the ratio to the wild-type. Error bars represent 95% confidence intervals. Similar patterns were found as in fitness values except in S→M and M→S where the fitness of ET1 and ET4 change significantly but the growth rates remain the same, when compared to the corresponding environment (M and S, respectively). The light blue dash line represents the product of fitness values for ET2 and ET4, which is not significantly different from the fitness values of ET1 in all environments. The result indicates ‘non-epistatic’ interaction between ET2 and ET4; the proportional effect of ET2 and ET4 is unchanged when present together in ET1. (TIF) [file pgen.1002651.s002.tif]

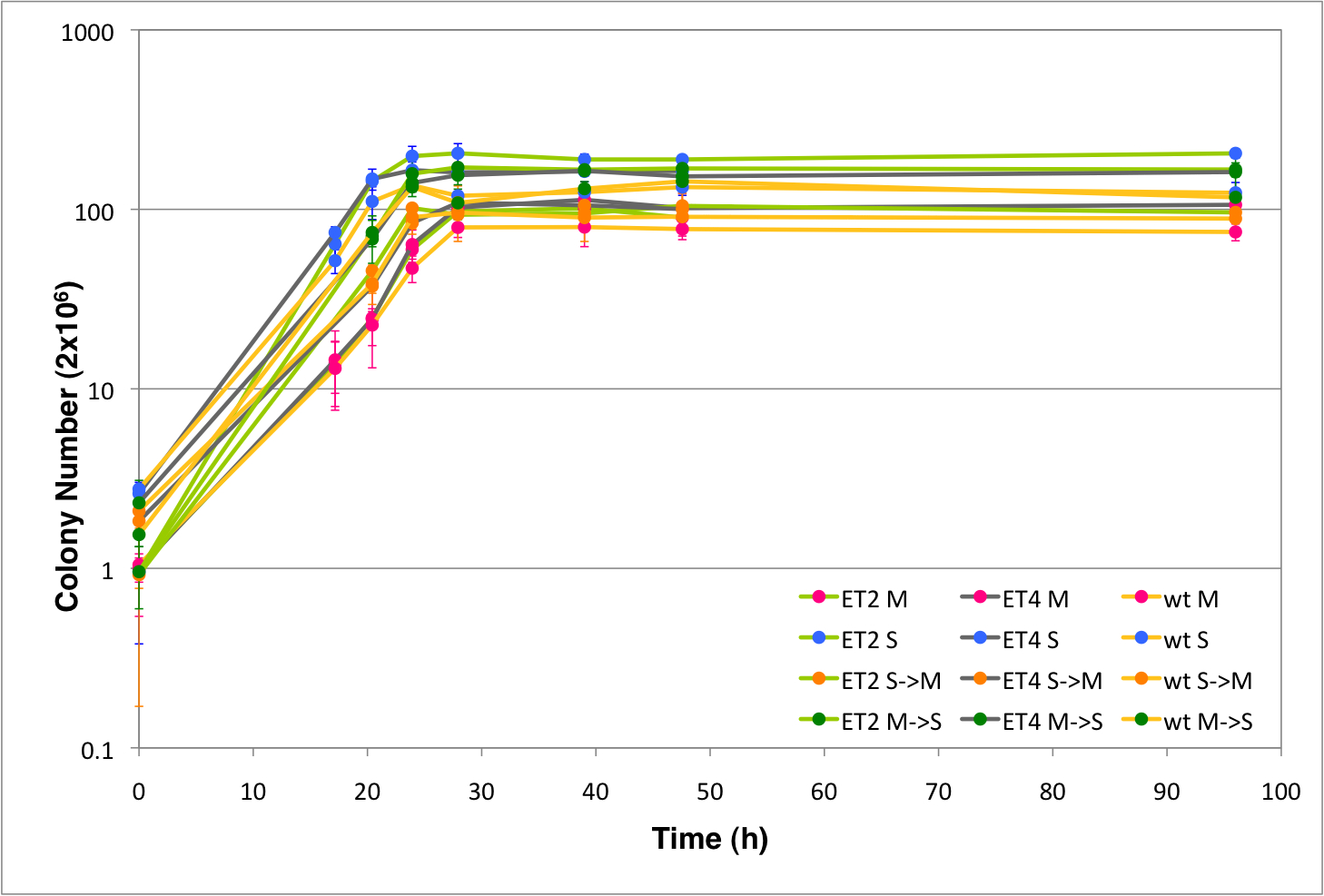

Supplement: Figure S3 — Viable counts of selected deletion mutants in 4 environments (M, S, M→S, S→M). All mutants and wild type have similar viable counts even after 96 hours. Less than 15% drop of viable counts was observed for all mutants. (TIF) [file pgen.1002651.s003.tif]

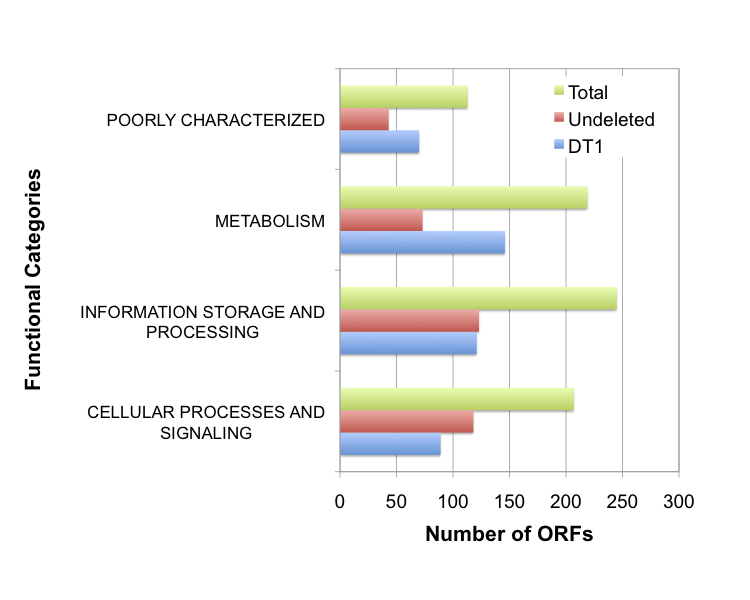

Supplement: Figure S4 — Functional analysis of DT1 versus undeleted region on the megaplsmid. The number of ORFs for each major functional category was calculated based on COG classification. There are substantially more metabolic related genes in DT1 region than the undeleted region. (TIF) [file pgen.1002651.s004.tif]

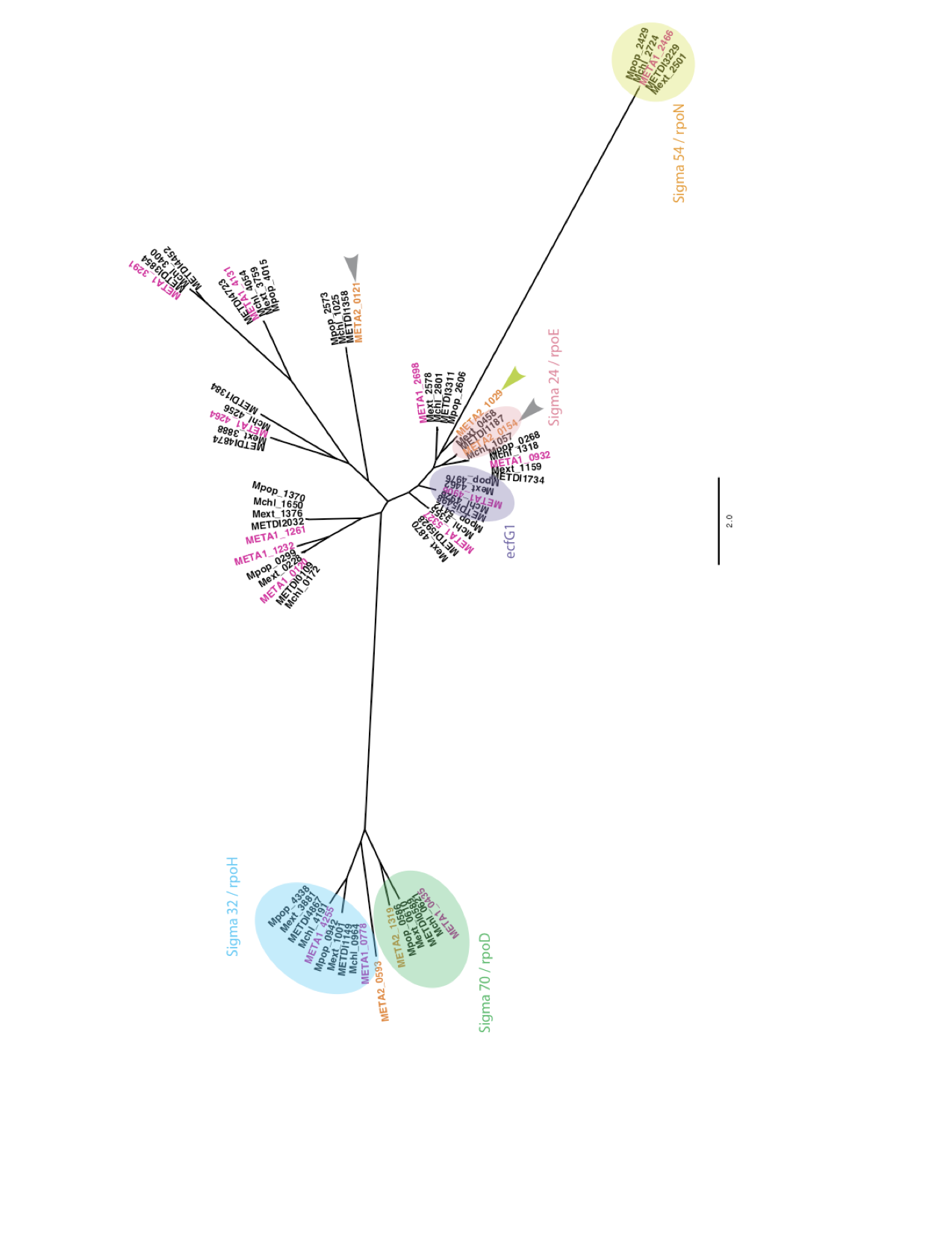

Supplement: Figure S5 — Phylogeny of sigma factors in M. extorquens AM1 (META1, pink text; META2, orange text) and closely related strains: M. extorquens DM4 (METDI), CM4 (Mchl), PA1 (Mext), M. populi BJ001 (Mpop). Five groups of sigma factors are colored based on the annotation. Arrows indicate the sigma factors which locate in the region of ET2 (green) and ET4 (grey). META2_0154 (ECF type) and META2_0121 (sigma 24) are conserved in all M. extorquens strains, suggesting a potential function of those two sigma factors. (TIF) [file pgen.1002651.s005.tif]
