# Supplementary material for: Repeated, Selection-Driven Genome Reduction of Accessory Genes in Experimental Populations
Source: PLoS Genet. 2012 May 10;8(5):e1002651. doi: 10.1371/journal.pgen.1002651 (PMC3349727; doi:10.1371/journal.pgen.1002651)
Supplement: Table S1 — Methylobacterium strains used in the study. (DOCX) [file pgen.1002651.s006.docx]

**Table S1.** *Methylobacterium* strains used in the study

| Strain | Source | Colony Morphology | Hybridization pair | Deletion region Chromosome  (META1) | Deletion region Megaplasmid  (META2) | Big deletion type determined by PCR^※^ |
| --- | --- | --- | --- | --- | --- | --- |
| CM501 | Ancestor | Pink | 3,4,25 | None | None | -- |
| CM502 | Ancestor | White | -- | None | None | -- |
| CM1203 | CM501, Δ*hprA* | Pink | 1 | None | None | -- |
| CM1027 | A1 | Pink, Large | -- | -- | -- | DT1a |
| CM1028 | A1 | Pink, Medium | 13 | 3908875~3921127* | 822917~844898 881288~206285^✝^ | DT1a |
| CM1029 | A1 | Pink, Small | -- | -- | -- | DT1a |
| CM1030 | A2 | White, Large | 21 | ND | 881288~206285^✝^ | DT1a |
| CM1031 | A2 | White, Medium | -- | -- | -- | DT1a |
| CM1032 | A2 | White, Small | 14 | ND | 838627~211269 | DT1a |
| CM1033 | A3 | Pink, Large | -- | -- | -- | DT1a |
| CM1034 | A3 | Pink, Medium | 15 | 3894244~3955912* | ND | ND |
| CM1035 | A3 | Pink, Small | -- | -- | -- | ND |
| CM1036 | A4 | White, Large | 16 | ND | 857393~168962 | DT1b |
| CM1037 | A4 | White, Medium | -- | -- | -- | DT1b |
| CM1038 | A4 | White, Small | -- | -- | -- | DT1b |
| CM1039 | A5 | Pink, Large | -- | -- | -- | ND |
| CM1040 | A5 | Pink, Small | -- | -- | -- | ND |
| CM1041 | A5 | Pink, Dark pink | 22 | ND | 855329~211852^✝^ | DT1a |
| CM1042 | A5 | Pink, Pale pink | 17 | ND | ND | ND |
| CM1043 | A6 | White, Large | 18 | ND | 881288~206285^✝^ | DT1a |
| CM1044 | A6 | White, Medium | 2 | ND | 281915~306778* 881288~206285^✝^ | DT1a |
| CM1045 | A6 | White, Small | -- | -- | -- | DT1a |
| CM1046 | A7 | Pink, Large | 19 | ND | ND | ND |
| CM1047 | A7 | Pink, Medium | -- | -- | -- | DT1a |
| CM1048 | A7 | Pink, Small | -- | -- | -- | DT1a |
| CM1049 | A8 | White, Large | -- | -- | -- | ND |
| CM1050 | A8 | White, Medium | 20 | ND | 826162~205962 | DT1a |
| CM1051 | A8 | White, Small | -- | -- | -- | DT1a |
| CM1086 | B1 | Pink, Large | -- | -- | -- | DT1a |
| CM1087 | B1 | Pink, Small | 13 | 3894244~3955912* | 881288~206285^✝^ | DT1a |
| CM1088 | B1 | Pink, Pale pink | -- | -- | -- | DT1a |
| CM1089 | B2 | White, Large | -- | -- | -- | DT1a |
| CM1090 | B2 | White, Medium | 14 | ND | 881288~206285^✝^ | DT1a |
| CM1091 | B2 | White, Small | -- | -- | -- | DT1a |
| CM1092 | B3 | Pink, Large | -- | -- | -- | DT1a |
| CM1093 | B3 | Pink, Medium | -- | -- | -- | DT1a |
| CM1094 | B3 | Pink, Small | 15 | ND | 881288~206285^✝^ | DT1a |
| CM1095 | B4 | White, Large | 16 | 4133302~4147632* | 855329~211852^✝^ | DT1a |
| CM1096 | B4 | White, Medium | -- | -- | -- | DT1a |
| CM1097 | B4 | White, Small | 2 | 4133302~4147632*  3894244~3955912* | 855329~211852^✝^ | DT1a |
| CM1098 | B5 | Pink, Small | 17 | 4133303~4155430* | 855329~211852^✝^ | DT1a |
| CM1099 | B5 | Pink, Dark pink | 21 | 3894244~3955912* 4133303~4155430* | 855329~211852^✝^ | DT1a |
| CM1100 | B5 | Pink, Pale pink | -- | -- | -- | DT1a |
| CM1104 | B6 | White, Large | 22 | ND | 881288~206285^✝^ | DT1a |
| CM1105 | B6 | White, Medium | 18 | ND | 881288~206285^✝^ | DT1a |
| CM1106 | B6 | White, Small | -- | -- | -- | DT1a |
| CM1107 | B6 | White, Small | -- | -- | -- | DT1a |
| CM1101 | B7 | Pink, Large | -- | -- | -- | DT2 |
| CM1102 | B7 | Pink, Medium | -- | -- | -- | DT2 |
| CM1103 | B7 | Pink, Small | 19 | ND | 883700~70551 | DT1b |
| CM1108 | B8 | White, Large | -- | -- | -- | DT1b |
| CM1109 | B8 | White, Medium | 20 | ND | 855329~211852^✝^ | DT1a |
| CM1110 | B8 | White, Small | -- | -- | -- | DT1a |
| CM1052 | C1 | Pink, Large | -- | -- | -- | DT2 |
| CM1053 | C1 | Pink, Medium | 23 | ND | 883700~1150049 | DT2 |
| CM1054 | C1 | Pink, Small | 3,5 | 2877651~2907157^✝^ 3894244~3955912* | 881288~206285^✝^ | DT1a |
| CM1055 | C2 | White, Large | 6 | 63646~80725 | 881288~206285^✝^ | DT1a |
| CM1056 | C2 | White, Large | -- | -- | -- | DT1a |
| CM1057 | C2 | White, Small | -- | -- | -- | DT1a |
| CM1058 | C2 | White, Small | -- | -- | -- | DT1a |
| CM1059 | C3 | Pink, Large | -- | -- | -- | DT1a |
| CM1060 | C3 | Pink, Medium | 7 | ND | 881288~206285^✝^ | DT1a |
| CM1061 | C3 | Pink, Small | -- | -- | -- | DT1a |
| CM1062 | C4 | White, Large | 24 | ND | 881288~206285^✝^ | DT1a |
| CM1063 | C4 | White, Medium | 8 | ND | 881288~206285^✝^ | DT1a |
| CM1064 | C4 | White, Small | -- | -- | -- | DT1a |
| CM1065 | C5 | Pink, Small | -- | -- | -- | DT1a |
| CM1066 | C5 | Pink, Dark pink | 9 | ND | 881288~206285^✝^ | DT1a |
| CM1067 | C5 | Pink, Pale pink | -- | -- | -- | DT1a |
| CM1068 | C6 | White, Large | 10 | 3894244~3955912* | 881288~206285^✝^ | DT1a |
| CM1069 | C6 | White, Medium | -- | -- | -- | DT1a |
| CM1070 | C6 | White, Small | -- | -- | -- | DT1a |
| CM1071 | C7 | Pink, Large | -- | -- | -- | DT1a |
| CM1072 | C7 | Pink, Medium | 11 | 4133299~4160613* | 881288~206285^✝^ | DT1a |
| CM1073 | C7 | Pink, Small | 25 | ND | 881288~206285^✝^ | DT1a |
| CM1074 | C8 | White, Large | 12 | 3894244~3955912* | 826162~205962 | DT1a |
| CM1075 | C8 | White, Medium | -- | -- | -- | DT1a |
| CM1076 | C8 | White, Small | -- | -- | -- | DT1a |
| CM1181 | D1 | Pink, Large | 4,5 | ND | ND | ND |
| CM1182 | D1 | Pink, Medium | -- | -- | -- | DT3b |
| CM1183 | D1 | Pink, Small | -- | -- | -- | DT2 |
| CM1184 | D2 | White, Large | -- | -- | -- | DT2 |
| CM1185 | D2 | White, Large | -- | -- | -- | DT2 |
| CM1186 | D2 | White, Small | 6 | ND | 303859~318885 838627~1150049 | DT2 |
| CM1187 | D3 | Pink, Large | -- | -- | -- | DT1b |
| CM1188 | D3 | Pink, Medium | 7 | ND | ND | ND |
| CM1189 | D3 | Pink, Small | 1 | ND | ND | ND |
| CM1190 | D4 | White, Large | -- | -- | -- | DT2 |
| CM1191 | D4 | White, Medium | 8 | ND | 868402~22943 | DT1b |
| CM1192 | D4 | White, Small | -- | -- | -- | DT1b |
| CM1193 | D5 | Pink, Small | 9 | ND | 857393~1150049 | DT2 |
| CM1194 | D5 | Pink, Dark pink | 23 | ND | 1118314~1141380 | DT3a |
| CM1195 | D5 | Pink, Pale pink | -- | -- | -- | DT2 |
| CM1820 | D6 | White, Large | 24 | 476704~485541 | 838627~142461 | DT1b |
| CM1821 | D6 | White, Medium | -- | -- | -- | DT1b |
| CM1822 | D6 | White, Small | -- | -- | -- | DT1a |
| CM1196 | D7 | Pink, Large | -- | -- | -- | ND |
| CM1197 | D7 | Pink, Large | 11 | ND | ND | ND |
| CM1198 | D7 | Pink, Small | -- | -- | -- | ND |
| CM1199 | D7 | Pink, Small | -- | -- | -- | ND |
| CM1200 | D8 | White, Large | 12 | ND | 883700~1150049 | DT2 |
| CM1201 | D8 | White, Medium | -- | -- | -- | ND |
| CM1202 | D8 | White, Small | 10 | ND | ND | ND |
| CM2074 | ET1 mutant of CM501 | pink | -- | None | 878552~210322 | ET1 |
| CM2075 | ET1 mutant of CM501 | pink | -- | None | 878552~210322 | ET1 |
| CM2076 | ET1 mutant of CM501 | pink | -- | None | 878552~210322 | ET1 |
| CM2077 | ET2 mutant of CM501 | pink | -- | None | 878552~1159793 | ET2 |
| CM2078 | ET2 mutant of CM501 | pink | -- | None | 878552~1159793 | ET2 |
| CM2079 | ET2 mutant of CM501 | pink | -- | None | 878552~1159793 | ET2 |
| CM2080 | ET4 mutant of CM501 | pink | -- | None | 1161223~210322 | ET4 |
| CM2081 | ET4 mutant of CM501 | pink | -- | None | 1161223~210322 | ET4 |
| CM2082 | ET4 mutant of CM501 | pink | -- | None | 1161223~210322 | ET4 |
| CM2083 | ET3 mutant of CM501 | pink | -- | None | 1112224~1159793 | ET3 |
| CM2084 | ET3 mutant of CM501 | pink | -- | None | 1112224~1159793 | ET3 |
| CM2085 | ET3 mutant of CM501 | pink | -- | None | 1112224~1159793 | ET3 |

* Deletion region was confirmed by PCR followed by sequence

^✝^ Deletion region was confirmed by PCR followed by restriction digestion

^※^ Deletion type was determined by four pairs of primers to amplify fragments: R1, R2, R3, R4 (see Table S3 for specific primer locations). DT1a: all 4 pairs gave negative results. DT1b: R1, R2, and R3 came back negative but R4 was positive. DT2: R1 and R2 came back negative but R3 and R4 were positive. DT3a: only one isolate has this deletion, which was detected by array. DT3b: R1, R2 and R4 came back positive but R3 was negative.
