# Supplementary material for: Repeated, Selection-Driven Genome Reduction of Accessory Genes in Experimental Populations
Source: PLoS Genet. 2012 May 10;8(5):e1002651. doi: 10.1371/journal.pgen.1002651 (PMC3349727; doi:10.1371/journal.pgen.1002651)
Supplement: Table S2 — Deletions detected by CGH arrays. (DOCX) [file pgen.1002651.s007.docx]

**Table S2.** Deletions detected by CGH arrays

| **Replicon** | **Region** | **Length** | **Strain** | **Adjacent IS^✝^** |
| --- | --- | --- | --- | --- |
| META1 | 63646~80725 | 17079 | CM1055 | ※ |
| META1 | 476704~485541 | 8837 | CM1820 | ※ |
| META1 | 2877651~2907157 | 29506 | CM1054 | ISMex15*2 |
| META1 | 3894244~3955912 | 61668 | CM1034, CM1087, CM1054, CM1068, CM1074, CM1099 | Repeat*2 |
| META1 | 3908875~3921127 | 12252 | CM1028 | ISMex16*0 |
| META1 | 4133302~4147632 | 14330 | CM1095^§B4^, CM1097^§B4^ | ISMex4*1 |
| META1 | 4133303~4155430 | 22127 | CM1098^§B5^, CM1099^§B5^ | ISMex4*1 |
| META1 | 4133299~4160613 | 27314 | CM1072 | ISMex4*1 |
| META2 | 281915~306778 | 24863 | CM1044 | ISMex16*0 |
| META2 | 303859~318885 | 15026 | CM1186 | ISMex8*2 |
| META2 | 826162~205962 | 641260 | CM1050, CM1074 | ISMex9*2 |
| META2 | 838627~1150049 | 311422 | CM1186 | ISMex5/ISMex10 *2 |
| META2 | 838627~142461 | 565294 | CM1820 | ISMex5/ISMex10 *1 |
| META2 | 838627~211269 | 634102 | CM1032 | ISMex5*2 |
| META2 | 822917~844898 | 21981 | CM1028 | ISMex9*1 |
| META2 | 855329~211852 | 617983 | CM1041, CM1095^§B4^, CM1097^§B4^, CM1098^§B5^, CM1099^§B5^, CM1109 | ISMex5*2 |
| META2 | 857393~1150049 | 292656 | CM1193 | ISMex5*1 |
| META2 | 857393~168962 | 573029 | CM1036 | ISMex5*1 |
| META2 | 868402~22943 | 416001 | CM1191 | ※ |
| META2 | 881288~206285 | 586457 | CM1028, CM1030, CM1043^§A6^, CM1044^§A6^, CM1054, CM1055, CM1060, CM1062^§C4^, CM1063^§C4^, CM1066, CM1068, CM1072^§C7^, CM1073^§C7^, CM1087, CM1090, CM1094, CM1104^§B6^, CM1105^§B6^ | ISMex3*2 |
| META2 | 883700~1150049 | 266349 | CM1053, CM1200 | ISMex3*1 |
| META2 | 883700~70551 | 448311 | CM1103 | ISMex3*1 |
| META2 | 1118314~1141380 | 23068 | CM1194 | ※ |

^✝^ Numbers after * represent the IS copy number in the ancestral genome. Ex: ISMex16*0 means the deletion was mediated by two newly inserted ISMex16.

※ No IS was found around the deletion but we have not ruled out the possibility that the deletion is mediated by newly inserted IS elements.

§ Identical deletions detected in isolates from the same population were counted as single event. In total, 46 deletion events were detected. Letters after § indicate the population number.
