# Supplementary material for: Repeated, Selection-Driven Genome Reduction of Accessory Genes in Experimental Populations
Source: PLoS Genet. 2012 May 10;8(5):e1002651. doi: 10.1371/journal.pgen.1002651 (PMC3349727; doi:10.1371/journal.pgen.1002651)
Supplement: Table S3 — Primer list. (DOCX) [file pgen.1002651.s008.docx]

**Table S3.** Primer list

|  | **Primer** | **Sequence** | **Replicon** | **Position** | | **Length** |
| --- | --- | --- | --- | --- | --- | --- |
| R1 | R1-f | CTT CGT CGA TTC AGC TCG TAC GT | META2 | 884409 | 884431 |  |
|  | R1-r | CCT GCA ACC AAG TCC TCT ACC ATC | META2 | 884817 | 884840 | 431 |
| R2 | R2-f | GGG TGC TTG GCA ATG TCT TAG GAA | META2 | 1115938 | 1115961 |  |
|  | R2-r | TTT CTT GCG TCT GTG CGA GCT TG | META2 | 1116295 | 1116317 | 379 |
| R3 | R3-f | TAA GCT TTC GCC TAA ACG CCT TCG | META2 | 1155765 | 1155788 |  |
|  | R3-r | ATT CAC CGA GAC TGT CCC AGA AGA | META2 | 1156189 | 1156212 | 447 |
| R4 | R4-f | ACA TTC GGT GGC AAC TCC TGA AG | META2 | 205547 | 205569 |  |
|  | R4-r | CCA CTG CGA CCG ATC TCT TAG TTC AT | META2 | 205965 | 205990 | 443 |
| PC | PC-f | CAG CTC GAC CAG CTT ATC GTT G | META1 | 4653445 | 4653466 |  |
|  | PC-r | ATC GTC TCC AAG TGC GGT G | META1 | 4654012 | 4653994 | 549 |
