# Supplementary material for: Repeated, Selection-Driven Genome Reduction of Accessory Genes in Experimental Populations
Source: PLoS Genet. 2012 May 10;8(5):e1002651. doi: 10.1371/journal.pgen.1002651 (PMC3349727; doi:10.1371/journal.pgen.1002651)
Supplement: Table S4 — Plasmid list. (DOCX) [file pgen.1002651.s009.docx]

**Table S4.** Plasmid list

| **Plasmid** | **Description** |
| --- | --- |
| pCM184 | Broad-host range cre-lox allelic exch, *Amp^R^, Kan^R^, Tet^R^ |
| pCM433 | Broad-host-range *sacB*-based allelic exchange vector, Amp^R^, Chl^R^, Tet^R^ |
| pML3 | pCM433 with META2_877176~878552, Amp^R^, Chl^R^, Tet^R^ |
| pML4 | pML3 with META2_210322~211783, Amp^R^, Chl^R^, Tet^R^ |
| pML5 | pML3 with META2_1159793~1161223, Amp^R^, Chl^R^, Tet^R^ |
| pML6 | pCM433 with META2_210322~211783, Amp^R^, Chl^R^, Tet^R^ |
| pML7 | pML6 with META2_1159793~1161223, Amp^R^, Chl^R^, Tet^R^ |
| pML8 | pCM433 with META2_1110807~1112224, Amp^R^, Chl^R^, Tet^R^ |
| pML9 | pML8 with META2_1159793~1161223, Amp^R^, Chl^R^, Tet^R^ |
| pML10 | pML4 with *loxP-Kan-loxP*, Amp^R^, Chl^R^, Tet^R^, Kan^R^ |
| pML11 | pML5 with *loxP-Kan-loxP*, Amp^R^, Chl^R^, Tet^R^, Kan^R^ |
| pML12 | pML7 with *loxP-Kan-loxP*, Amp^R^, Chl^R^, Tet^R^, Kan^R^ |
| pML13 | pML9 with *loxP-Kan-loxP*, Amp^R^, Chl^R^, Tet^R^, Kan^R^ |

* Amp (ampicillin), Chl (chloramphenicol), Kan (kanamycin), Tet (tetracycline)
