# Supplementary material for: Repeated, Selection-Driven Genome Reduction of Accessory Genes in Experimental Populations
Source: PLoS Genet. 2012 May 10;8(5):e1002651. doi: 10.1371/journal.pgen.1002651 (PMC3349727; doi:10.1371/journal.pgen.1002651)
Supplement: Table S5 — Functional gene list within deletion region. (DOCX) [file pgen.1002651.s010.docx]

**Table S5.** Functional gene list within deletion region*

| **Gene ID** | **Begin** | **End** | **Length** | **Gene** | **Product** |
| --- | --- | --- | --- | --- | --- |
| META2_0021 | 34645 | 37704 | 3060 | *nrd* | Ribonucleoside-diphosphate reductase |
| META2_0052 | 62867 | 64453 | 1587 |  | Beta-lactamase family protein |
| META2_0064 | 72260 | 72817 | 558 |  | Signal receiver domain protein |
| META2_0084 | 89378 | 92524 | 3147 |  | Heavy metal efflux pump, CzcA family |
| META2_0101 | 104280 | 105572 | 1293 |  | Conserved hypothetical protein, putative Type II site-specific deoxyribonuclease |
| META2_0113 | 112567 | 115731 | 3165 |  | Heavy metal efflux pump, CzcA family |
| META2_0121 | 120692 | 121300 | 609 |  | RNA polymerase ECF-type sigma factor |
| META2_0140 | 134208 | 135620 | 1413 | *pyk* | Pyruvate kinase |
| META2_0142 | 136331 | 137077 | 747 | *arsH* | Arsenate resistance protein ArsH |
| META2_0143 | 137128 | 137487 | 360 | *arsR2* | Transcriptional regulator, ArsR family, ArsR2 |
| META2_0144 | 137480 | 138007 | 528 | *arsC3* | Arsenate reductase |
| META2_0145 | 138007 | 138432 | 426 | *arsC* | Arsenate reductase |
| META2_0147 | 138708 | 139508 | 801 |  | Arsenite efflux pump ACR3 (fragment) |
| META2_0150 | 141288 | 143048 | 1761 |  | Peptidase S8 and S53, subtilisin, kexin, sedolisin precursor |
| META2_0154 | 143577 | 144257 | 681 |  | RNA polymerase sigma-24 subunit, ECF subfamily |
| META2_0155 | 144738 | 146327 | 1590 |  | Filamentation induced by cAMP protein Fic |
| META2_0159 | 147891 | 148055 | 165 |  | Cytochrome *c* biogenesis protein, transmembrane region precursor (fragment) |
| META2_0163 | 150286 | 151521 | 1236 |  | Carbohydrate-selective porin OprB |
| META2_0168 | 154237 | 154971 | 735 |  | DNA-binding response regulator in two-component regulatory system |
| META2_0169 | 154968 | 156302 | 1335 |  | Periplasmic sensor signal transduction histidine kinase |
| META2_0174 | 159624 | 162872 | 3249 | *czcA2* | Heavy metal efflux pump CzcA |
| META2_0176 | 163478 | 164395 | 918 | *czcD* | Cation efflux system protein CzcD |
| META2_0200 | 179043 | 179231 | 189 |  | Cytochrome biogenesis protein (fragment) |
| META2_0202 | 180534 | 181055 | 522 |  | Signal peptidase II |
| META2_0203 | 181052 | 183247 | 2196 | *zntA* | Zinc, cobalt and lead efflux system |
| META2_0211 | 188773 | 191922 | 3150 | *czcA* | Heavy metal efflux pump CzcA |
| META2_0214 | 194332 | 194544 | 213 | *copP* | CopP metal-binding protein, putative exported protein |
| META2_0217 | 196175 | 199348 | 3174 | *cusA* | Copper/silver efflux system, membrane component |
| META2_0940 | 887992 | 888432 | 441 | *trxC* | Thioredoxin |
| META2_0962 | 907338 | 908885 | 1548 |  | DNA primase |
| META2_0971 | 913402 | 916275 | 2874 | *polA* | DNA polymerase I |
| META2_0992 | 930257 | 932395 | 2139 | *ligA* | DNA ligase, NAD(+)-dependent |
| META2_0995 | 934201 | 935235 | 1035 | *ruvB* | ATP-dependent DNA helicase, component of RuvABC resolvasome |
| META2_1007 | 941539 | 942450 | 912 |  | Beta-lactamase domain protein |
| META2_1009 | 943278 | 943727 | 450 |  | Predicted transporter component |
| META2_1015 | 948261 | 948923 | 663 |  | DNA-binding response regulator in two-component regulatory system |
| META2_1016 | 948920 | 950236 | 1317 |  | Integral membrane sensor signal transduction histidine kinase |
| META2_1021 | 953252 | 955546 | 2295 | *katG* | Catalase/hydroperoxidase HPI(I) |
| META2_1023 | 956106 | 957488 | 1383 |  | Sensor histidine kinase |
| META2_1024 | 957481 | 958158 | 678 |  | DNA-binding response regulator in two-component regulatory system |
| META2_1026 | 959725 | 962862 | 3138 | *czcA2* | RND divalent metal cation efflux transporter CzcA |
| META2_1029 | 963246 | 963947 | 702 |  | RNA polymerase ECF-type sigma factor |
| META2_1058 | 986951 | 987994 | 1044 |  | Tryptophanyl-tRNA synthetase (Tryptophan--tRNA ligase) (TrpRS) |
| META2_1077 | 1004463 | 1004873 | 411 |  | HNH endonuclease family protein |
| META2_1083 | 1010052 | 1012508 | 2457 | *secA* | Preprotein translocase SecA |
| META2_tRNA1 | 1013193 | 1013282 | 90 |  | Ser tRNA |
| META2_1086 | 1013881 | 1014990 | 1110 |  | 3'-phosphoadenosine 5'-phosphosulfate sulfotransferase sulfate assimilation) |
| META2_1098 | 1027261 | 1029525 | 2265 |  | Protein with a domain similar to DNA-directed DNA polymerases |
| META2_1117 | 1048329 | 1049405 | 1077 |  | Nitrilase |
| META2_1120 | 1051831 | 1052604 | 774 |  | Nitrilase |
| META2_1122 | 1052823 | 1053830 | 1008 | *qxtB* | Cytochrome bd-quinol oxidase subunit II |
| META2_1124 | 1055306 | 1056532 | 1227 | *hemT* | 5-aminolevulinate synthase |
| META2_1127 | 1058729 | 1059643 | 915 | *tehA* | Potassium-tellurite ethidium and proflavin transporter |
| META2_1128 | 1059679 | 1060185 | 507 |  | Cytochrome *c* family protein |
| META2_1133 | 1062849 | 1063358 | 510 |  | Ion transport domain protein, C-terminal |
| META2_1134 | 1062862 | 1063626 | 765 |  | Iron transport domain protein, N-terminal |
| META2_1135 | 1063853 | 1065067 | 1215 | *nhaA* | Sodium-proton antiporter |
| META2_1141 | 1068358 | 1069020 | 663 | *uhpA* | DNA-binding response regulator in two-component regulatory system with UhpB |
| META2_1190 | 1100140 | 1105398 | 5259 |  | DEAD/DEAH box helicase domain protein |
| META2_1191 | 1105395 | 1108154 | 2760 |  | Helicase domain protein |
| META2_1211 | 1126021 | 1127037 | 1017 |  | NADPH quinone oxidoreductase, Zinc-containing alcohol dehydrogenase superfamily |
| META2_1213 | 1127541 | 1128443 | 903 |  | Transcriptional regulator, LysR family |
| META2_1218 | 1131773 | 1132627 | 855 |  | Transcriptional regulator, LysR family |
| META2_1225 | 1137855 | 1138760 | 906 |  | Helix-turn-helix, AraC type:AraC-type transcriptional regulator |
| META2_1226 | 1138897 | 1139640 | 744 |  | Short-chain dehydrogenase/reductase |
| META2_1230 | 1141030 | 1141839 | 810 | *phnC* | Phosphonate transport protein (ABC superfamily) |
| META2_1233 | 1143570 | 1144589 | 1020 | *ptxD* | Phosphonate dehydrogenase (NAD-dependent phosphite dehydrogenase) |
| META2_tRNA2 | 1162482 | 1162555 | 74 |  | Gln tRNA |
| META2_1291 | 1186946 | 1187614 | 669 | *rnhB* | Ribonuclease HII, degrades RNA of DNA-RNA hybrids |
| META2_1296 | 1189815 | 1190708 | 894 |  | Homolog of eukaryotic DNA ligase III |
| META2_1331 | 1221864 | 1225304 | 3441 |  | Chromosome segregation-like (SMC) protein |

* Excluding all hypothetical proteins, putative enzymes, and transposases.
