# Supplementary material for: Repeated, Selection-Driven Genome Reduction of Accessory Genes in Experimental Populations
Source: PLoS Genet. 2012 May 10;8(5):e1002651. doi: 10.1371/journal.pgen.1002651 (PMC3349727; doi:10.1371/journal.pgen.1002651)
Supplement: Text S1 — (DOCX) [file pgen.1002651.s011.docx]

**Repeated, selection-driven genome reduction of accessory genome in experimental populations**

Ming-Chun Lee,^1^ & Christopher J. Marx^1,2,*^

Supporting Information

Text S1

# Adaptation in selective environments at generation 1500

Three or four strains were isolated from each population at generation 1500 and their fitness was tested against the fluorescent-labeled ancestor as described in Materials and Methods. The average fitness of each environment was calculated and shown in Figure S1. Overall, the average fitness of evolved populations increased by 15% to 37% in their respective selective environments, indicating the adaptation has occurred in those populations.

**Deletion dynamics**

By applying the PCR technique as described in methods to the whole population samples at different time points, we found DT1 fixed in 4 populations (B2, B3, B5, B6) before generation 900 and in an additional 9 populations (A1, B1, B4, C3-C8) before generation 1500. We also screened a limited number of available isolates from B populations at generation 480, 540, and 720 (each with ~8 isolates), and found no isolate from generation 480 and 540 had a deletion. However, at generation 720, over 30% of the isolates already had DT1. Interestingly, over 50% of the B isolates had DT2 at this time point, but DT1 was present at generation 1500. The results suggest either DT1 happened in a stepwise fashion, or there might have been multiple genotypes in the population and the multiple DT lineages coexisted due to clonal interference.

**Fitness effect and growth rate of deletion mutants**

To examine the phenotypic effects of these deletions, we reconstructed four types of compatible deletion mutants (ET1, 2, 3 and 4) (Figure 2A) under the ancestral background by allelic exchange using *sacB* and *cre-lox* systems (1, 2), which let us eliminate the confounding effects of other mutations which occurred along the evolutionary experiment. Figure 2B shows the fitness effects of each of the deletion types across the 4 environments: M, S, MS, and M/S (the average of M🡪S & S🡪M). The simplest results were from ET2 where the fitness is slightly beneficial with significant fitness increase of 2 to 4 % in all environments (*P* < 0.0001) except in M🡪S where significance is marginal (*P* = 0.0603) and from ET3 where the fitness is nearly neutral with increases of 0 to 1% in all environments (*P* range from 0.01 to 0.0001), indicating ET2 and ET3 are generically either slightly beneficial or neutral across all environments in the ancestral background. We hypothesize, however, that they may be beneficial in later backgrounds due to epistasis. On the other hand, we found strong environmental specific fitness effects from ET1 and ET4 (Figure 2B). In single substrate environments, substantial fitness increases on S were found both from ET1 (*W* = 1.145, *P* < 0.0001) and ET4 (*W*= 1.107%, *P* < 0.0001), while the fitness effects on M were rather mild (ET1: *W* = 1.026, *P* = 0.0002; ET4: *W* = 0.984, *P* = 0.0027) and ET4 is actually deleterious when growing in M-only environment. In the combined-nutrient environments (MS and M/S), we found the fitness of ET1 and ET4 were consistent with the null expectation of being close to the average of the fitness values from the two single-substrate environments (Figure 2B). However, in the alternating environment, there was a substantial fitness decrease on M when transferring from S (~4%, *P* < 0.0001), but an increased fitness on S when transferring from M (~7%, *P* < 0.0001), relative to the single substrate environments (Figure 2A). This suggested a substantial effect of the transition between nutrients in both ET1 and ET4. Furthermore, we found the product of fitness values from ET2 and ET4 is very close to the fitness of ET1 across all environments (Figure S2), indicating no deviance in epistasis from independence, such that the transition effect stems from ET4. This result further supports the hypothesis that the region of ET4 was important for nutrient switching.

To get a better understanding of the underlying causes of the fitness changes when switching environments, we performed growth analyses of the engineered deletion mutants. The results revealed a clear discrepancy between the growth rate and fitness values for ET1 and ET4 (Figure S2). In both cases we found the relative growth rates of ET1 and ET4 on either substrate was indistinguishable whether the previous growth cycle was on M or S, indicating growth rate was not the reason for the fitness changes. Due to the limitation on sensitivity of measuring OD_600_ at the low initial values, tracking lag phase itself was fairly inaccurate and did not allow observation of significant differences in the duration of the lag phases between wild type and mutants. However, taking the advantage of the natural diauxic growth observed when *M. extorquens* AM1 switches from S to M, we uncovered a significantly longer transition time from S to M both in ET1 and ET4 (ET1: *P* = 0.0008; ET4: *P* = 0.0078) (Figure 2C), which strongly supports the hypothesis of fitness effect of these deletions on nutrient switching. In addition, ET1 and ET4 also exhibited a significantly higher fitness cost during stationary phase (*P* < 0.0001) (Figure 2D).

The long transition from S to M explains the fitness drop, and the fitness cost at stationary phase might explain the fitness difference between S and M🡪S in view of the fact that M🡪S culture experiences shorter stationary phase due to longer lag phase when switching substrates. However, this disadvantage during stationary phase was not due to the survival rate when growing alone. The viable count did not change appreciably (less than 15%) even after 96 hours for all strains (Figure S3). Alternatively, this disadvantage appeared to be due to a small degree of differential cryptic growth during stationary phase.

**Gene content**

Of the 606 coding sequences in the region of DT1, 415 are annotated as conserved/hypothetical proteins without further informative functions. The major groups of the remaining 191 genes include genes associated with mobile elements, regulatory domains, and stress responses (Table S5). Comparative genomics analysis with two closely related *Methylobacterium extorquens* strains DM4 (NC_012988) (3) and CM4 (NC_011757) also revealed that most of the genes of the megaplasmid are unique to AM1. The only region shared with the other strains of the species is a region around the end of DT1 (Figure 1A) that is present on the chromosome of strain DM4 and CM4, suggesting the potential importance of this region. Interestingly, we found two beta-lactamase family proteins related to ampicillin resistance and one operon related to arsenate resistance located on the region of DT1, although both of them have other homologs on the main chromosome. Moreover, there are 5 sigma factors on the megaplasmid and 3 of them are in the region of DT1. We further performed phylogenetic analysis of those sigma factors along with other sigma factors in the closely related *Methylobacterium* strains and found two of them do not have any homolog in the main chromosome but are conserved in other *Methylobacterium* strains (Figure S5). One of those two sigma factors is a homolog of σ^24^ (or RpoE) and the other is an ECF type sigma factor, which are associated with heat and stress resistance (4). Additionally, we also found 5 genes in the region of DT1 to be induced during phyllosphere colonization (5), suggesting a potential tradeoffs in the ability to associate with plants due to this deletion type. These analyses also revealed a potential replication origin in the region just upstream of DT1, possibly explaining why the boundary of the genome reduction did not extend farther.

**Stress responses**

Although our data clearly indicate a selective advantage of these deletions under their respective selective conditions, we were interested in extending our phenotypic analysis to investigate the possibility of further tradeoffs in alternative environments (*i.e.*, antagonistic pleiotropy). Our analysis of the gene content lost in the deletions, particularly the ET4 region, pointed to the possibility of altered stress responses. Consistent with the finding of two beta-lactamase family proteins and one operon related to arsenate resistance locating on the region of ET4 (both of them have other homolog genes on the main chromosome), we found that the sensitivity of ET1 and ET4 increased significantly in media with either ampicillin (12.5 µg/mL) (Figure 2E) or arsenate (30mM) (Figure 2F). Finding genes with possible involvement in heat shock (σ^32^ homolog and protein folding/degradation functions) led us to hypothesize deletions of the ET4 region would lead to increased sensitivity to high temperatures. Surprisingly, we found the opposite: a significant improvement on growth rate at 36 °C from ET1 and ET4 (Figure 2G) but the cause of this improvement is still unknown. Other general stresses tested that failed to reveal differences compared to wild-type included: formaldehyde, SDS, peroxide, metal mix, salt, heat shock or UV treatments, as well as all disc diffusion assays. Still, the decrease of antibiotic and heavy metal resistance in liquid medium indicated a tradeoff of this genome reduction.

**Lack of epistasis between ET2 and ET4**

All phenotypes tested for ET1 and ET4 were qualitatively the same, other than slight differences in the magnitude of effect. This small difference appears to be easily accounted for by including the phenotypic effect of the other half of the large ET1 deletion: ET2. Thus, although the ET1 phenotype is largely due to the ET4 region, the ET1 phenotype could be accounted for by considering each half-deletion to maintain the proportional effect on phenotype that was observed when present alone. The fact that we never found deletions similar to ET4 in the naturally evolved populations and the rare occurrence of DT1 in M/S environment, and the fitness cost during the S🡪M switch raise suggests that a gene (or genes) within this region contributes to nutrient switching. Furthermore, the fact ET4 was never found in populations despite sometimes being strongly beneficial (e.g., 10% on S) could be due to epistatic interactions with other mutations that had already occurred within those lines that either reduced or eliminated this selective advantage.

**Mutation accumulation**

We compared our findings from the evolved populations to those from a series of 10 mutation accumulation populations of the identical *M. extorquens* AM1 transferred through single-colony bottlenecks for 1500 generations. From a total of 10 x 1,500 = 15,000 generations across the populations, no deletions of any type in the region of DT1 were observed. On the other hand, the net rate we observed across the large *N_e_* populations is at least 11.55 per 15,000 generations (37 PCR-detectable deletions across 32 x 1,500 = 48,000 generations assuming the deletions did not occur until exactly 1500 generations). The *P*-value of 0 events in 15, 000 generation under the Poisson distribution of this net rate would be less than 0.0001 by applying the following formula:


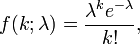
 , where λ=11.55, and *k*=0.

The actual difference is even greater, given that the mutations occurred prior to 1500 generations in order to have been observed by then. These data indicate that the observed parallelism here was due primarily, if not exclusively, to selection.

#

**References**

1. Marx CJ & Lidstrom ME (2002) Broad-host-range *cre-lox* system for antibiotic marker recycling in gram-negative bacteria. *Biotechniques* 33(5):1062-1067.

2. Marx C (2008) Development of a broad-host-range *sacB*-based vector for unmarked allelic exchange. *BMC Res Notes* 1(1):1.

3. Vuilleumier S*, et al.* (2009) *Methylobacterium* genome sequences: a reference blueprint to investigate microbial metabolism of c1 compounds from natural and industrial sources. *PLoS ONE* 4(5):e5584.

4. Hiratsu K, Amemura M, Nashimoto H, Shinagawa H, & Makino K (1995) The *rpoE* gene of *Escherichia coli*, which encodes sigma E, is essential for bacterial growth at high temperature. *J Bacteriol* 177(10):2918-2922.

5. Gourion B, Rossignol M, & Vorholt JA (2006) A proteomic study of *Methylobacterium extorquens* reveals a response regulator essential for epiphytic growth. *Proc Natl Acad Sci USA* 103(35):13186-13191.
